# Randomized Controlled Study Comparing Efficacy and Toxicity of Weekly vs. 3-Weekly Induction Chemotherapy in Locally Advanced Head and Neck Squamous Cell Carcinoma

**DOI:** 10.3389/fonc.2020.01284

**Published:** 2020-08-06

**Authors:** Devale Tousif, Vinu Sarathy, Rajesh Kumar, Radheshyam Naik

**Affiliations:** ^1^HealthCare Global Enterprises Ltd (HCG), Bangalore, India; ^2^HCG Cancer Hospital, Bengaluru, India

**Keywords:** induction chemotherapy, weekly docetaxel, weekly cisplatin in head and neck cancer, locally advanced head and neck cancer, weekly 5-fluro uracil, weekly TPF

## Abstract

**Background:** Head and Neck Cancer is a major public health problem in India, majority of which are lifestyle related, male predominant requiring dedicated infrastructure and human resource. The 5-year survival is 59% for all stages combined and only 45% in patients with locally advanced inoperable head and neck cancer using current chemoradiation schedules. Chemotherapy agents administered in the induction or concurrent setting comprise of taxanes (Docetaxel, paclitaxel), platinum compounds (Cisplatin, carboplatin) and fluorouracil (TPF). For patients with advanced Head and neck squamous cell carcinoma (HNSCC), 3-weekly TPF regimen is the established standard induction chemotherapy (ICT) option based on overall survival benefit. However, TPF regimen is known to be associated with significant dose limiting toxicities which may impair tolerance and effectiveness of therapy. In this study we assessed the efficacy and toxicity of weekly vs. 3-weekly Docetaxel, Cisplatin, and Fluoro-uracil (TPF) induction chemotherapy in locally advanced Head and neck squamous cell carcinoma (LA-HNSCC).

**Methods:** This was an open labeled randomized two arm study with 41 patients in the 3-weekly TPF arm and 41 patients in the weekly arm. Patients were randomized using numbers from a randomization software, data recorded, and results were analyzed.

**Results:** The weekly group achieved far greater symptom relief than 3-weekly group (72 vs. 64%). The overall response rates were similar in both arms (ORR 75.6 and 73.1% in the weekly and 3-weekly groups, respectively). Renal toxicity was significantly lower in the weekly group as compared to 3 weekly arm post three cycles of chemotherapy (CrCl 91.49 ml/min vs. 76.67 ml/min, respectively). The weekly group had predominantly grade I and II neutropenia (19.5 and 17.1%, respectively) as compared to 3-weekly group where grade III and IV neutropenia (31 and 12%, respectively) was more prominent (*p*-0.003). Among non-hematological toxicities, mucositis, nausea/vomiting, and diarrhea in the weekly group were significantly lower when compared to 3-weekly group. Progression free survival was slightly higher in the weekly group (18 months) when compared to 3-weekly group (15 months) which was not statistically significant.

**Conclusion:** Weekly induction with TPF had lower toxicity and similar efficacy as compared to 3-weekly regimen in locally advanced HNSCC patients. Myelosuppression, which was the most serious and common complication of 3-weekly TPF regimens was notably low using the weekly regimen. Our results suggest that weekly TPF regimen may be a safer and effective alternative to 3-weekly TPF for treatment of LA-HNSCC. To our knowledge this is the first study reporting the efficacy of weekly TPF regimen in LA-HNSCC till date.

## Introduction

Head and Neck Cancers (HNCs) comprise of malignancies of the oral/nasal cavity, lips, salivary glands, pharynx (hypo pharynx, oropharynx and nasopharynx), and larynx. Squamous cell carcinomas (SCCs) constitute ~90% of Head and Neck Cancers with adenocarcinomas, melanomas, and sarcomas forming the rest (1). Head and Neck Cancers are emerging as a major health problem in India. Overall 57.5% of global head and neck cancers occur in Asia out of which around 30–35% occur in India (2). The 5-year relative survival rate is 81% for patients with localized disease and 59% for all stages combined (3, 4).

To improve response rates and functional outcomes, chemotherapy has been included into various schedules. Chemotherapy agents with proven activity in squamous cell carcinoma commonly used in either induction or concurrent chemotherapy regimens consist of the platinum compounds (Cisplatin, carboplatin), fluorouracil, and taxanes (Docetaxel, paclitaxel). The three-drug combination of platinum, fluorouracil plus taxane is the preferred regimen for induction chemotherapy (ICT). Randomized trials found that addition of a taxane (Docetaxel, paclitaxel) to PF regimen improved efficacy of induction chemotherapy (5, 6). The most extensive data on TPF regimen comes from the TAX 324 trial, in which 501 patients were randomly assigned to induction with Docetaxel, Cisplatin, plus fluorouracil (TPF) or PF followed by concurrent chemo radiotherapy using weekly carboplatin. Although TPF regimen had an improved overall survival than PF, TPF regimen was also associated with significant acute toxicities and myelosuppression (7, 8).

We hypothesized that weekly induction chemotherapy with TPF regimen may have similar efficacy and lower toxicity further improving tolerability and response rates. In this study we assessed the efficacy and toxicity of weekly vs. 3-weekly Docetaxel, Cisplatin, and Fluoro-uracil (TPF) induction chemotherapy in locally advanced Head and neck squamous cell carcinoma (HNSCC).

## Materials and Methods

The study was conducted in HealthCare Global Hospital, Bangalore after approval from institutional ethics committee. The study was a prospective two arm open labeled randomized controlled study which included locally advanced HNSCC (LA-HNSCC) patients recruited during 1st April 2015 to 31st March 2017. The study included a total of 82 LA-HNSCC patients. After taking informed consent the patients were randomized into two groups; Group A and Group B (41 patients in each group), to receive 3 cycles of weekly and 3-weekly TPF regimen, respectively. Randomization was done using random numbers generated by the software (www.randomizer.org) (9).

### Treatment Protocol

The following treatment protocol was used for patients allotted to GROUP A or B.

**Group A**: Patients in this group received weekly chemotherapy for 9 weeks.

1. Docetaxel-30 mg/m^2^ i/v infusion on D1
2. Cisplatin-40 mg/m^2^ i/v infusion on D1
3. 5FU-750 mg/m^2^ i/v infusion over 6 h on D1

**Group B:** Patients in this group received three-weekly chemotherapy for 3 cycles (from D1 to D5).

1. Docetaxel-75 mg/m^2^ i/v infusion on D1
2. Cisplatin-75 mg/m^2^ i/v infusion on D1
3. 5-FU-750 mg/m^2^ i/v infusion over 24 h from D1-D5

### Response Evaluation

Radiological Response assessment was done by Response Evaluation Criteria in Solid Tumors RECIST (version 1.1) based on PET scan imaging modality. Clinical response grading was done according to National Cancer Institute- Common Terminology Criteria for Adverse Events (NCI- CTCAE) version 4.0 (10).

### Statistical Methods

SPSS version 23 was used for data analysis. Frequencies and percentage were reported for categorical variables. Continuous variables were expressed as mean and standard deviation for normally distributed data and median and range for skewed data. Kaplan Meier survival analysis was carried for progression free survival. Patient characteristics were evaluated by using Chi-Square test. Results are graphically represented where deemed necessary. Probability values below 0.05 were considered as statistically significant.

## Results

### Patient's Characteristics

The patients' baseline characteristics are summarized in Table 1. Majority of patients were between 51 and 60 years of age in both the study groups. Both the groups had a male preponderance. It is a well-known fact that co-morbid conditions are associated with poorer tolerance and response rates, thus being a major confounding factor in the study. Most patients in both groups of our study did not have any major co-morbidity. The few patients with co-morbid conditions were well-controlled during the study period. All the three variables were well-matched in our study (Table 1).

**Table 1:** Patient baseline characteristics of the two groups.

**Variable**	**Weekly TPF (%)**	**3-weekly TPF (%)**	***p*-value**
**Age (mean ± SD)**			
21–30	2 (4.9)	1 (2.4)	0.263
31–40 years	4 (9.8)	10 (24.4)	
41–50 years	7 (17.1)	10 (24.4)	
51–60 years	28 (68.3)	20 (48.8)	
**Gender**			
Male	33 (80.5)	35 (85.4)	0.345
Female	8 (19.5)	6 (14.6)	
**Comorbidities^1^**			
Nil	28 (68.3)	30 (73.2)	0.872
DM (Grade 1)	8 (19.5)	5 (12.2)	
HTN (Grade 1 and 2)	4 (9.8)	5 (12.2)	
IHD (Grade 1)	1 (2.4)	1 (2.4)	
**Subsite**			
Oropharynx	26	25	0.601
Hypopharynx	15	15	
Oral cavity	0	1	
**Performance status (ECOG)**			
1	40	41	0.314
2	1	0	
**HPV – p16**			
Oropharynx	5	4	0.532
Hypopharynx	2	1	
**Symptoms**			
Dysphagia			0.587
Grade 1	0	0	
Grade 2	0	0	
Grade 3	21	19	
Grade 4	8	7	
Pain			
Grade 1	0	0	
Grade 2	0	0	
Grade 3	8	7	
Hoarseness			
Grade 1	0	0	
Grade 2	0	1	
Grade 3	4	7	
Total	41 (100)	41 (100)	

1 *Available online at: https://www.rtog.org/LinkClick.aspx?fileticket=oClaTCMufRA%3D&tabid=290 (accessed June 15, 2020)*.

### Assessment of Clinical Symptoms

Severity of clinical symptoms before and after chemotherapy in the weekly and 3-weekly groups is summarized in Table 2. Clinical symptoms of patients were recorded as per NCI-CTCAE Version 4.0.

**Table 2:** Grade of clinical symptoms - Pre and Post chemotherapy in Weekly and 3-Weekly groups.

**Grade of clinical symptoms**	**At presentation**				**Post chemotherapy**			
	**Weekly**		**3-weekly**		**Weekly**		**3-weekly**	
Grade 1	-	-	-	-	27	65.9	17	41.5
Grade 2	0	0	1	2.4	11	26.8	18	43.9
Grade 3	33	80.5	33	80.5	3	7.3	6	14.6
Grade 4	8	19.5	7	17.1	-	-	-	-
Total	41	100	41	100	41	100	41	100

#### Pre Chemotherapy

In both groups GR III symptoms were more common. The weekly group had 80.5 and 19.5% of GR III and IV clinical symptoms, respectively, while the 3-weekly group had 2.4, 80.5 and 17.5% of GR II, III, and IV symptoms, respectively.

#### Post Chemotherapy

Overall better symptom relief was achieved in the weekly group as compared to 3-weekly group. The weekly group showed a 72% reduction of grade III clinical symptoms as opposed to 64% reduction in the 3-weekly group.

### Radiological Responses

**Figure 1:**
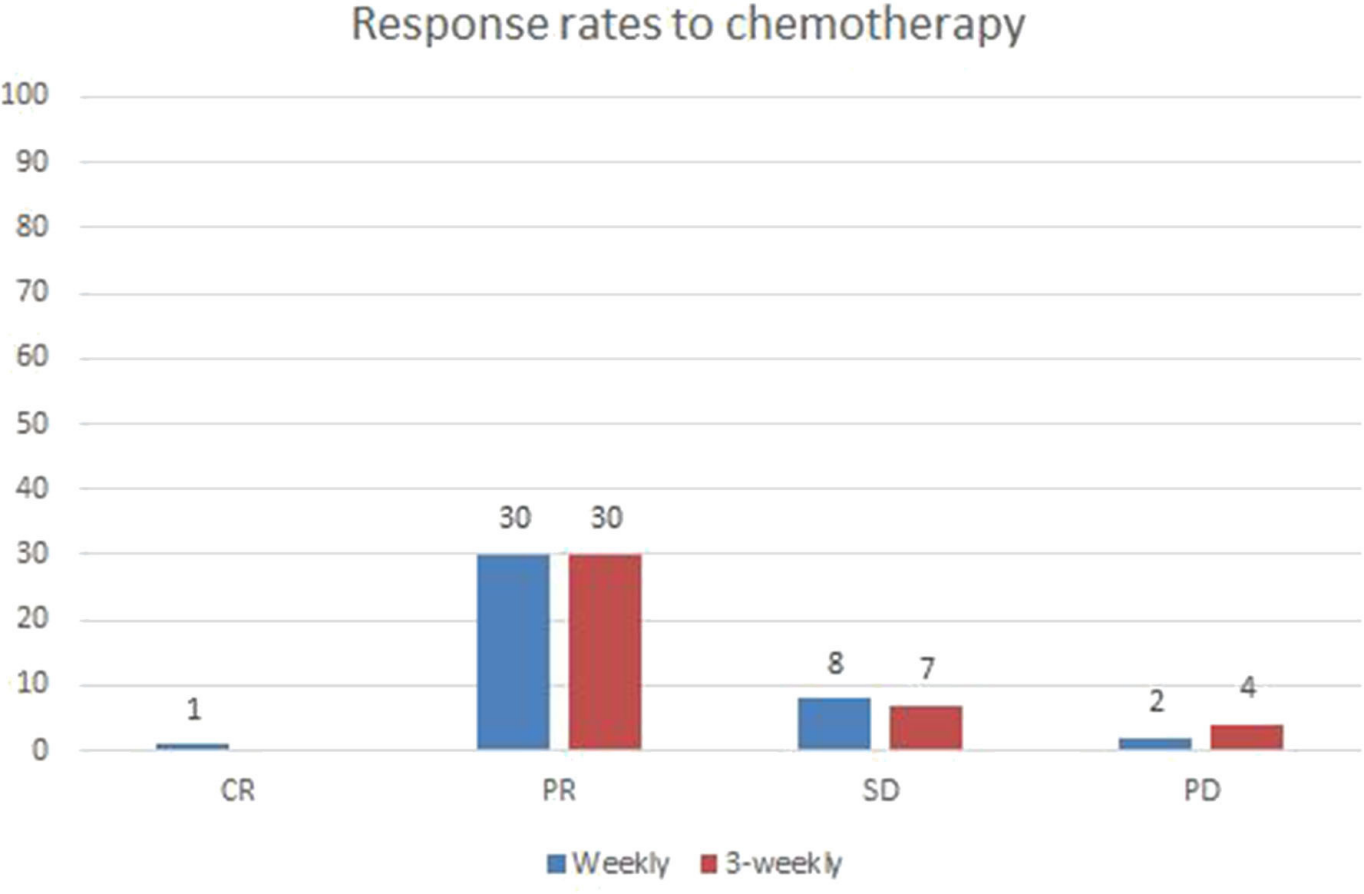
Radiological Responses:—Post chemotherapy in both groups.

Post treatment radiological responses are represented in Figure 1. Radiological response was evaluated using RECIST criteria (1.1). Overall response rate (ORR) included complete and partial responses. The weekly group had an ORR of 75.6 and an almost similar ORR of 73.1% was seen in 3-weekly group (*p*-0.687). The number of patients with stable and progressive disease were also similar between the two groups.

### Evaluation of Toxicities in the Weekly and 3-Weekly Group

The hematological and non-hematological toxicities in both groups are summarized in Table 3.

**Table 3:** Details of toxicity observed in weekly and 3-weekly groups.

**Details**	**Neutropenia**				**Mucositis**				**Nausea/vomiting**				**Diarrhea**				**Neuropathy**			
	**Weekly**		**3-Weekly**		**Weekly**		**3-Weekly**		**Weekly**		**3-Weekly**		**Weekly**		**3-Weekly**		**Weekly**		**3-Weekly**	
	***N***	**%**	***N***	**%**	***N***	**%**	***N***	**%**	***N***	**%**	***N***	**%**	***N***	**%**	***N***	**%**	***N***	**%**	***N***	**%**
Grade-I and II	15	36.6	13	31.7	26	63.4	12	29.3	22	53.7	11	26.8	8	19.6	21	51.2	8	19.5	15	36.6
Grade III	1	2.4	5	12.2	4	9.8	18	43.9	3	7.3	24	58.5	0	0	4	9.8	0	0	1	2.4
Grade IV	2	4.9	13	31.7	0	0	11	26.8	0	0	6	14.6	0	0	0	0	0	0	0	0
Nil	23	56.1	10	24.4	11	26.8	0	0	16	39	0	0	33	80.5	16	39	33	80.5	25	61
Total	41	100	41	100	41	100	41	100	41	100	41	100	41	100	41	100	41	100	41	100

The weekly group had more grade I and II neutropenia (19.5 and 17.1%, respectively) while the 3 weekly group had predominantly grade III and IV neutropenia (31 and 12%, respectively) which was statistically significant (*p*-0.003). Most of the patients in the weekly group tolerated ICT well without any major hematological adverse events.

The weekly ICT group had predominantly a lower grade of mucositis which included GR II and I (34 and 29%, respectively) as compared to 3-weekly group who had more of GR III and IV mucositis (9 and 26%, respectively; *p*-0.003). 26% of weekly-ICT group had no episode of mucositis. Higher grades of nausea and vomiting were seen in the 3-weekly group (58 and 14% in GR III and IV, respectively) while majority of weekly group had no symptoms (39%) or lower grades of nausea/vomiting (31 and 22% in GR II and GR I, respectively) with significant *p*-value (0.000). High grade diarrhea was seen chiefly in the 3-weekly group (36 and 9% GR II and III, respectively) while majority of weekly group patients had minimal or no symptoms (80%), *P*-value (0.001). Majority of patients in both groups tolerated chemotherapy well with mainly incidences of grade I (19% in each group) neurological symptoms in both groups (*p*-0.028) (Table 3).

### Nephropathy

**Figure 2:**
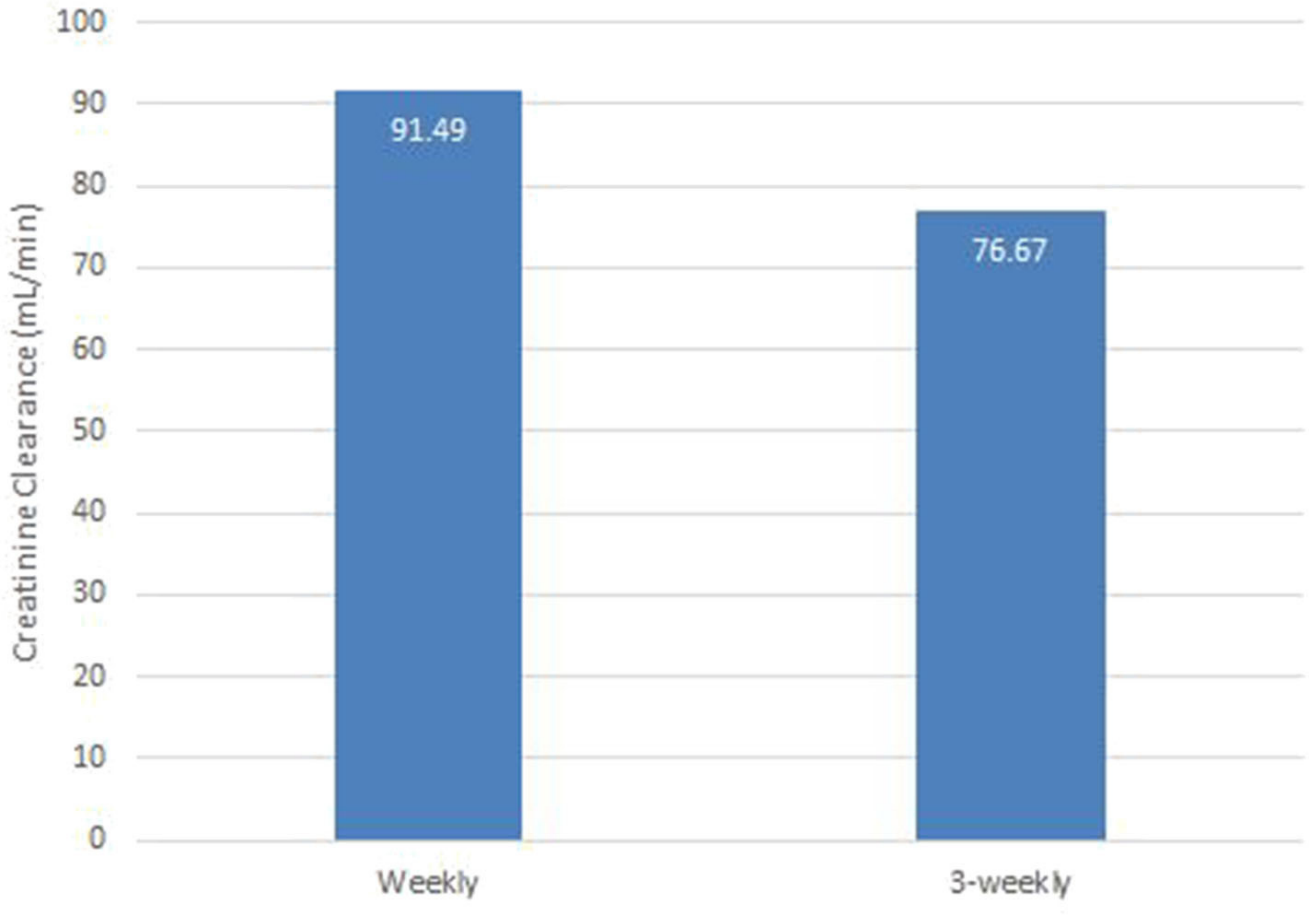
Nephropathy; Post chemotherapy in both groups.

Renal toxicity was analyzed using creatinine clearance (CrCl). The mean CrCl in weekly and 3-weekly groups was 91 and 76 ml/min, respectively (*p*-0.003). The weekly group had better renal function than 3-weekly group at the end of 3 cycles of chemotherapy. Assessment of renal function post chemotherapy is illustrated in Figure 2.

### Survival Analysis

**Figure 3:**
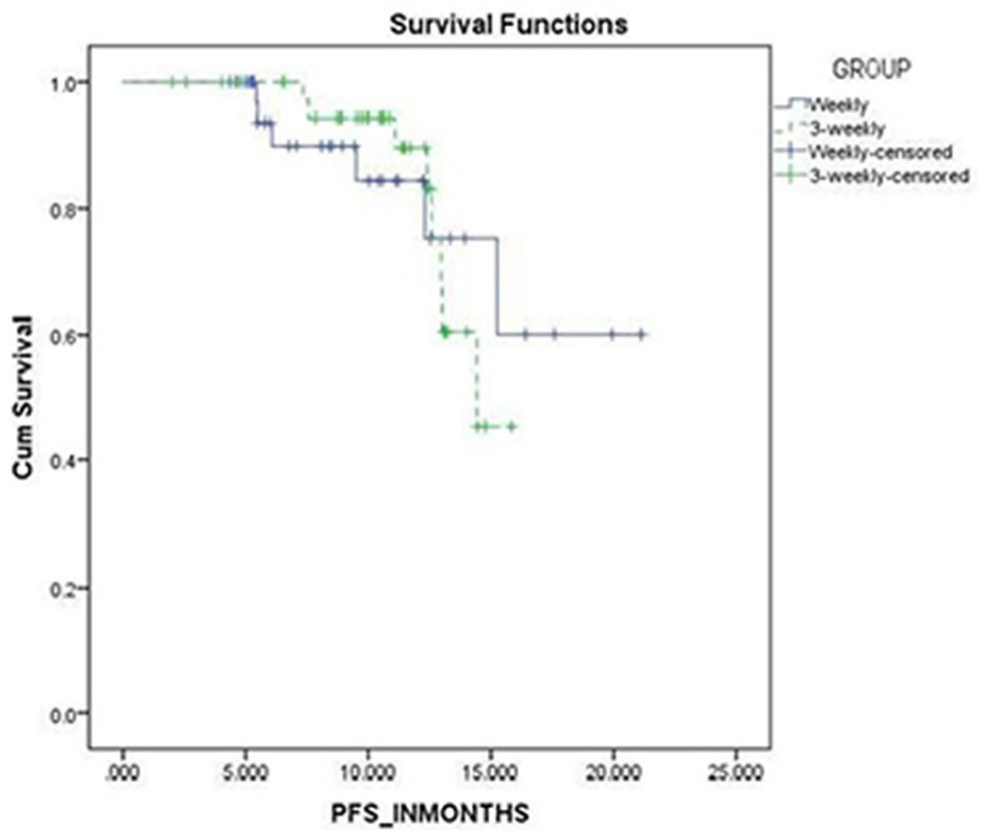
Kaplan Meier analysis of progression free survival.

The progression free survival was 18 and 15 months in the weekly and 3-weekly groups, respectively which was not statistically significant (*p* = 0.905) (Figure 3).

## Discussion

Induction chemotherapy has been mentioned as a first line option especially in oropharyngeal and hypopharyngeal locally advanced SCC (11). This has been demonstrated effectively in the landmark TAX-323 and TAX-324 trials (7, 8).

Posner et al., reported longer overall and progression free survival and a non-significant reduction of toxicities in the TPF group as compared with PF (7). However, this study also reported more myelo-suppression in the TPF group (83%) as compared to PF (56%). In the TAX 323 and TAX 324 studies, the main toxicity associated with TPF regimen was leukopenia and neutropenia indicating a clear need for regimens with improved tolerability and lower toxicity (12). Hence our study was designed to establish an effective, safe and tolerable daycare regimen using TPF as ICT for locally advanced HNSCC.

The early phase I/II trials of 3-weekly TPF in advanced HNSCC where docetaxel given at 75 mg/m^2^ every 3 weeks showed 95% grade 3–4 neutropenia and 19% febrile neutropenia (13). Rapidis et al. conducted a study aiming at reduction of myelosuppression and reported that using biweekly Docetaxel (40 mg/m^2^) with Cisplatin and 5-FU significantly decreased grade 3–4 neutropenia to 37% (14). This response suggested that biweekly or weekly Docetaxel could be an effective alternative for the 3-weekly regimen. Kean f, HO et al. demonstrated more delays (29 vs. 41%) and omission of chemotherapy (5.6 vs. 17.4%) occurred in 3-weekly arm as compared to the weekly group. They concluded that 100 mg/m^2^ of Cisplatin given every 3-weekly with radiotherapy was much less tolerated than 40 mg/m^2^ administered weekly and hence fewer patients achieved the effective cumulative dose of >200 mg/m^2^, possibly compromising on efficacy (15). A retrospective study by Patil et al. used induction chemotherapy with weekly paclitaxel and carboplatin in patients with LA-HNSCC and found that the above regimen was safe and effective even in elderly and those with poor performance status (16). Recently, the Japanese trial found that weekly cisplatin+RT is non-inferior to 3-weekly cisplatin+RT in LA-SCCHN pts and has a favorable toxicity profile, though this was reported in adjuvant setting (17). This formed the rationale for our study comparing standard 3-weekly with modified weekly TPF regimen using docetaxel at a dose of 30 mg/m^2^, Cisplatin 40 mg/m^2^ and 5-FU 750 mg/m^2^, all administered weekly for 9 weeks followed by re-assessment. To our knowledge this is the first studying comparing weekly TPF induction with standard 3-weekly regimen in locally advanced head and neck squamous cell cancer.

## Cumulative Dose Intensity and its Significance

The weekly group received a higher cumulative dose of taxanes and platinum by 16.7 and 37.5%, respectively at the end of 3 weeks as compared to the 3-weekly group. Whereas, the cumulative dose of 5-FU was 40% lower in the weekly group when compared to 3-weekly group. Despite higher dose intensity achieved for docetaxel and cisplatin in the weekly group, hematological and non-hematological toxicity was predominantly only grade I or II.

At presentation, the weekly group mainly had GR III and IV clinical symptoms in 80.5 and 19.5%, respectively while the 3-weekly group presented with GR II, III, and IV symptoms in 2.4, 80.5, and 17.5%, respectively. Post 3 cycles, the weekly group showed greater relief in symptoms with objective reduction from clinical grade III to grade I as compared to the 3-weekly group. The above effect was close to significant (*p* = 0.084). The response rate (RR) was almost similar in both groups with an ORR of 75.6% in weekly group and 73.1% in the 3-weekly group. Difference in hematological toxicities was analyzed and the weekly group had more grade I and II neutropenia (19.5 and 17.1%, respectively) as compared to 3-weekly group where grade III and IV neutropenia (31 and 12%, respectively) was more significant. The above observation was statistically significant (*p*-0.003). Among the non-hematological toxicities: mucositis, nausea/vomiting and diarrhea were significantly lower in the weekly group as compared to 3-weekly group.

A single arm retrospective study done at TATA Memorial Hospital, Mumbai in 2014 by Patil et al., reported an overall response rate (CR + PR) of 67% (10 patients) (16). Overall grade 3–4 toxicity was seen in 6 patients. No toxicity related mortality was observed. The median PFS and OS were 10.36 and 16.53 months, respectively. Similarly, our study reported an ORR (CR + PR) of 75.6% (31 patients) in the weekly group. Only 2 patients had grade 3–4 toxicity. Summarizing, the weekly TPF group had significantly lower incidences of both hematological and non-hematological toxicities as compared to 3-weekly group.

The progression free survival was 18 and 15 months in the weekly and 3-weekly groups, respectively which was not statistically significant (*p* = 0.905).

The proposed mechanisms are that the dose dense approach facilitates for constant exposure of taxane to cells in G2-M phase thus preventing emergence of resistance clones and enhancing anti-tumor effect. Furthermore, weekly taxane may have direct angiogenic effects disrupting microtubule dynamics in the endothelial cells. This was particularly seen at a cytostatic concentration <10 nm. Weekly taxane may theoretically also reverse the resistance acquired to 3-weekly taxanes (18). Weekly taxanes may also be beter tolerated as myelosuppression depends on peak plasma concentration >50 nm. Hence weekly paclitaxel may decrease myelosuppression, maintain dose intensity and quicker rates of plasma concentration decline could limit toxicity (19, 20). Similarly regardless of treatment regimen, it has been suggested that a cumulative dose of 200 mg/m^2^ needs to be reached for therapeutic benefit in cisplatin studies (21, 22). A retrospective analysis indicated an inferior outcome with a cumulative cisplatin dose of ≤200 mg/m^2^ in HPV-negative patients. In our study the weekly cisplatin arm received a much higher cumulative dose (360 mg/m^2^) than the 3-weekly arm (225 mg/m^2^) possibly contributing to increased efficacy. Short 5-FU infusional schedules have also been reportedly used with considerable success in advanced head and neck cancers (23, 24).

The limitation of our study is small sample size. Assessment of HPV status was done by p16 Immunohistochemistry and not by Polymerase Chain Reaction (PCR). Quality of life post ICT was not recorded which may have helped in further analysis of effectiveness. Assessment of chronic toxicity was not part of our study protocol which can help to assess quality of life in long term survivors.

## Conclusion

Weekly TPF combination as an ICT showed significantly lower toxicity and similar efficacy as 3-weekly regimen in locally advanced HNSCC patients. Myelosuppression, which was the most serious and common complication of 3-weekly TPF regimen was notably low using the weekly regimen. Our results suggest that a weekly TPF regimen represents a safer and effective alternative to 3-weekly TPF for the treatment of LA-HNSCC. Further large-scale studies with longer follow up are needed to assess survival and long term toxicities using weekly TPF regimen in locally advanced head and neck cancers.

## Data Availability Statement

The raw data supporting the conclusions of this article will be made available by the authors, without undue reservation.

## Ethics Statement

The studies involving patients with locally advanced squamous cell carcinoma of head and neck were reviewed and approved by HCG-Central Ethics Committee bearing register no. ECR/386/INST/KA/2013. The patients/participants provided their written informed consent to participate in this study.

## Author Contributions

All authors listed have made a substantial, direct and intellectual contribution to the work, and approved it for publication.

## Conflict of Interest

VS was employed by HealthCare Global Enterprises Ltd. The remaining authors declare that the research was conducted in the absence of any commercial or financial relationships that could be construed as a potential conflict of interest.
